# Dual-Phase Lock-In Amplifier Based on FPGA for Low-Frequencies Experiments

**DOI:** 10.3390/s16030379

**Published:** 2016-03-16

**Authors:** Gonzalo Macias-Bobadilla, Juvenal Rodríguez-Reséndiz, Georgina Mota-Valtierra, Genaro Soto-Zarazúa, Maurino Méndez-Loyola, Mariano Garduño-Aparicio

**Affiliations:** Laboratorio de Mecatrónica, Universidad Autónoma de Querétaro, Cerro de las Campanas, Col. Las Campanas, S/N, Querétaro 76010, Mexico; gonzalo.macias@uaq.mx (G.M.-B.); georgina.mota@uaq.mx (G.M.-V.); genaro.soto@uaq.mx (G.S.-Z.); carmaury@hotmail.com (M.M.-L.), dr.mariano.g@ieee.org (M.G.-A.)

**Keywords:** Field-Programmable-Gate-Array, Phase-Shift-Detection, Lock-In-Amplifier, frequency-stability

## Abstract

Photothermal techniques allow the detection of characteristics of material without invading it. Researchers have developed hardware for some specific Phase and Amplitude detection (Lock-In Function) applications, eliminating space and unnecessary electronic functions, among others. This work shows the development of a Digital Lock-In Amplifier based on a Field Programmable Gate Array (FPGA) for low-frequency applications. This system allows selecting and generating the appropriated frequency depending on the kind of experiment or material studied. The results show good frequency stability in the order of 1.0 × 10^−9^ Hz, which is considered good linearity and repeatability response for the most common Laboratory Amplitude and Phase Shift detection devices, with a low error and standard deviation.

## 1. Introduction

Usually, there are two types of processes to study the response of a system that interacts with an Alternating Current (AC) signal. One of those is the Frequency Scan, where the outcoming signal is read from a range of frequencies. The other one is called Time Scan. In this case, the frequency is constant, and the outcoming signal is time-dependent. For a specific application, the range of frequencies depends on the response of the system and the detector bandwidth, among others. For example, in Photothermal and Photoacoustic Spectroscopy, it is common to make low-frequency and time scans from 10 Hz–10 kHz. However, in Photothermal Radiometry (PTR) and Photocarrier Radiometry (PCR), the common frequencies scans are from 10 kHz to 100 kHz, to study thermal and electronic properties, as well as some predetermined frequency values to obtain Photothermal and Photocarrier images at determined frequency [1,2,3]. 

The detection of these Photothermal signals is frequently made using Phase Sensitive Detection (PSD) to detect a characteristic of the signal (Amplitude and Phase) from a modulated system. PSD is also one of the fundamental operations that Lock-In Amplifiers (LIAs) use to reject undesired signals, excluding the stimulation signal [4]. 

The PSD process can be done using either analog or digital methods. The analog form uses an analog multiplier to make a convolution process that is an intrinsic feature in PSD. The multiplier of analog Lock-in Amplifiers (LIAs) is one of the most expensive elements. The better the precision and quality of the multiplication operation required, the more hardware requirements there are and the higher the cost becomes. On the other hand, the digital process uses a discrete multiplier to perform the convolution operation, which is cheaper when compared to the analog multiplier. The precision is directly related to the digital resolution of the multiplier. Another variable of an LIA consists of the range of frequencies that can be handled by its main oscillator [4,5,6,7]. 

For example, the range of frequencies used in applications as Photoacoustic, or some Photothermal radiometry experiments, is lower than 10 kHz. Rodríguez *et al*. [3] developed a new differential photoacoustic cell, in his experimental setup used two LIAs working at frequencies lower than 1 kHz. Recently, Velazquez-Hernandez *et al*. [2] and Rojas-Rodríguez *et al*. [1] published studies on thermal images that were carried out at 10 kHz frequency [1,2,3].

Photothermal techniques allow the detection of both qualitative and quantitative characteristics with a non-invasive method. Many researchers have reported on photothermal experiments employing commercial LIAs, but most of them only used basic functions [8,9,10,11,12,13,14,15,16,17].

Researchers have developed hardware for some specific Lock-In applications. Barragan, *et al*. [5] presented a Lock-in based on a Digital Signal Processor (DSP) for optical attenuation measurement; his implemented device may work in a 0–20 kHz range. Rastelli [7] developed a Field Programmable Gate Array (FPGA) based Lock-in for high frequencies, in this case, the specific purpose was photon counting. The aforementioned confirms that PSD development for some specific application is very important. It also reduces some problems, such as large space needs, unnecessary electronic functions, and high hardware costs, among others [5,7].

The purpose of the works above mentioned, it is to show that not always is necessary to have an expensive Lock-In device with many complex functions since the experiments required just use one frequency or a narrow frequency range. Besides a reconfigurable digital PSD (main unit of an LIA) is an easier and cheaper implementation in most of the low-frequency experiments. In this work, the optimized and internal multipliers from an FPGA circuit (reconfigurable element) were used to develop and to implement this signal processor. Previously, an analog to digital conversion of the measured signal is done when experiments are running. 

Furthermore, the designed device allows the generation and selection of the appropriated frequencies, depending on the kind of experiment or material handled, from 1 Hz up to 10 kHz. The developed reconfigurable system is based on FPGA, and may be implemented with a wide frequency range, depending on the Analog to Digital Converter (ADC) and the Digital to Analog Converter (DAC) used for implementation. The developed FPGA core can also be used for others experiments at higher frequencies. Also, it may be applied for on-line systems or permanent monitoring systems with low-cost elements, and implemented with a minimum amount of support resources. Essentially this eliminates the need of purchasing an expensive device that mostly is not used to its full capacity. Some tests, resolution experiments and comparison of results with commercial LIAs have presented this work herein to demonstrate the operation ranges and resolution for low-frequency applications. 

## 2. Principles of the Digital Lock-In 

The operational principle of analog LIAs is widely documented. It consists of the reading of a modulated signal (*V_read_*), which has a known frequency, and then it is multiplied by a reference signal (*V_ref_*) of the same frequency. This reference may be either a square or a sine form signal. As a result, of this multiplication or convolution process, a new signal (*V_result_*) of double frequency is obtained; and it is offset by the read signal variations (Equation (1)). The average (offset) of this resultant signal is related to both phase shift and amplitude variations of read signal (Equation (2)).

(1) Vread=Asigsin(ωrt+θsig)Vref=Arefsin(ωrt+θref)Vresult=Vread*VrefVresult=12AsigArefcos{(ωr−ωr)t+θsig−θref}      −12AsigArefcos{(ωr+ωr)t+θsig−θref}

(2) Vaverage result=12AsigArefcos(θsig−θref)

Because these obtained signals have both variations (amplitude and phase) inherently, dual-phase LIAs is commonly used as a double reference (Equation (3)). The second reference signal is shifted +90° concerning the first one. If the first reference (*V_ref1_*) is a sinusoidal signal, then the second reference is a cosine signal (*V_ref2_*). The average of the convolution result between read signal and sinusoidal (*V_ref1_*) reference is called *X*, and the average of the convolution of read signal and cosine reference (*V_ref2_*) is called Y. Finally, to compute amplitude (*R*) and phase (θ) is necessary to calculate the square root of (*X^2^ + Y^2^*) (Equation (4)) and the inverted tangent of *Y/X*, respectively (Equation (5)).

(3) Vread=Asigsin(ωrt+θsig)Vref1=Arefsin(ωrt+θref)Vref2=Arefcos(ωrt+θref)X=Vaverage result1=12AsigArefcos(θsig−θref)Y=Vaverage result2=12AsigArefsin(θsig−θref)

(4) $$R=\sqrt{\left(X^{2}+Y^{2}\right)}$$

(5) $$\theta =\tan^{-1}(Y/X)$$

In the case of digital LIAs, a similar process is made. The main difference is that an acquired signal is converted to digital data before the convolution process is made, and the internal reference is digital too. An Analog to Digital Converter (ADC) is used to convert the read signal. Obviously, the converted resolution is related to the application requirements or the desired precision for the experiment. The internal reference consists of a sine or cosine waveform generated by an internal algorithm that may be stored in a Look-Up Table (LUT). As in the case of read signal, the reference signal resolution is also related to the specific applications or the desired precision. 

To make the convolution process, digital LIAs employs a digital multiplier instead of an analog one in contrast to the analog LIAs. The captured signal is multiplied one point at a time and passed to a filter module. Digital multipliers are cheaper than analog multipliers, reducing the cost of the digital Lock-In development. 

As Equation (3) shows, the average of resultant signals (*X = V _average result1_* and *Y = V _average result2_*) is related to the phase shift and the amplitude variations of acquired signal. A low pass filter is used to obtain this average, and to remove the AC components. In this work, a specialized digital module has been used to obtain the average of resultant signals. Finally, given *X* and *Y*, to calculate Amplitude (*R*) and Phase (θ) is necessary to implement the mathematical operations in Equations (4) and (5) [7].

## 3. Hardware Requirement in Low-Frequency Photoacoustic Applications 

**Figure 1:**
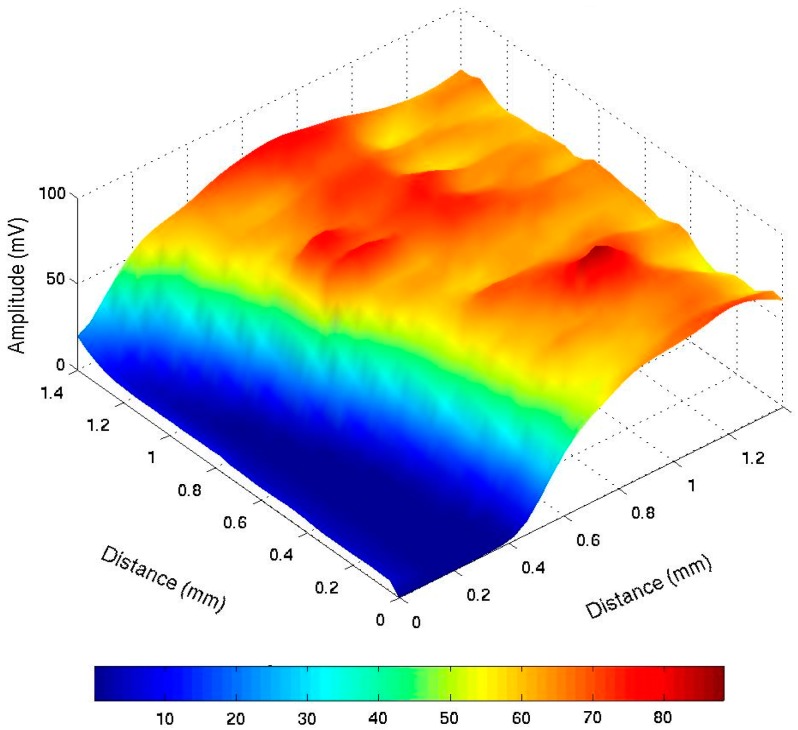
PTR amplitude response from steel 1018 at 10 Hz–1 KHz.

**Figure 2:**
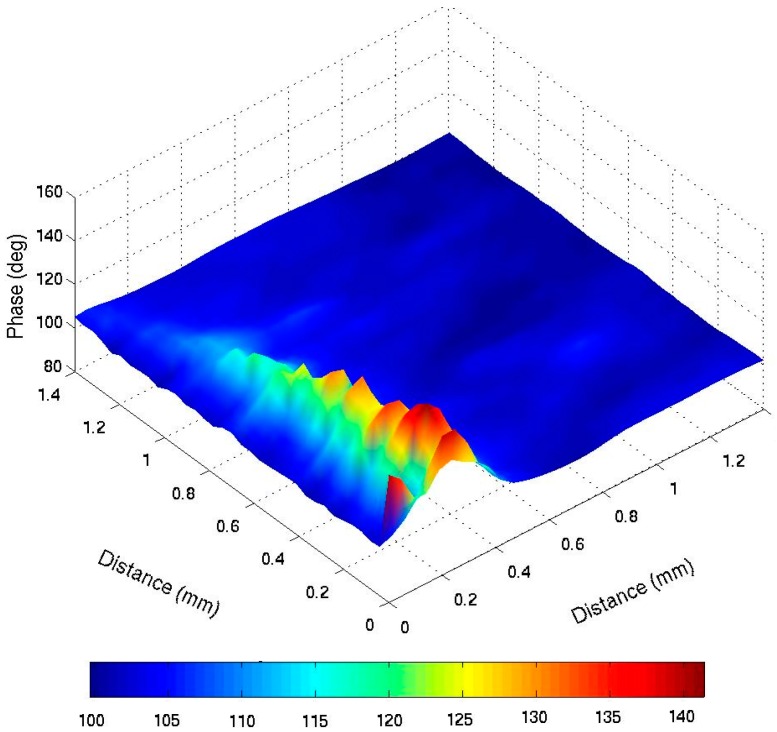
PTR phase response from steel 1018 at 10 Hz–1 KHz.

As mentioned before, many Photothermal experiments show results at low frequencies ranging from 1 Hz to 10 kHz, which includes variations of amplitude and phase. Figure 1 shows PTR results from steel 1018 response at 10 Hz–1 kHz, where amplitude variations can be observed clearly between thermal properties from the material. Figure 2 shows phase response from the same material, where phase changes up to 40° can be observed. Figure 1 and Figure 2 are just an example, and are similar to the result observed in previous publications by Velazquez-Hernandez *et al*., Rojas-Rodríguez *et al.*, and Rodríguez *et al*. [1,2,3] at different materials.

These examples show that important photothermal activity occurs at low frequencies, depending on the kind of experiment. This activity may appear in amplitude or phase variations. 

It is important to mention that results may differ from one experimental setup to another, which means that the amplitude results may vary, even in the same kind of experiment, if an external amplifier or laser intensity is modified. In this case, the most important issue is to observe the same behavior from the studied material and not the same numeric values. A similar variation may occur in phase response due to the experimental setup modifications. 

Different kind of experiments exists that show important activity response at low frequencies that are fully documented in the literature. 

## 4. Reconfigurable Hardware Design and Frequency Stability Test

**Figure 3:**
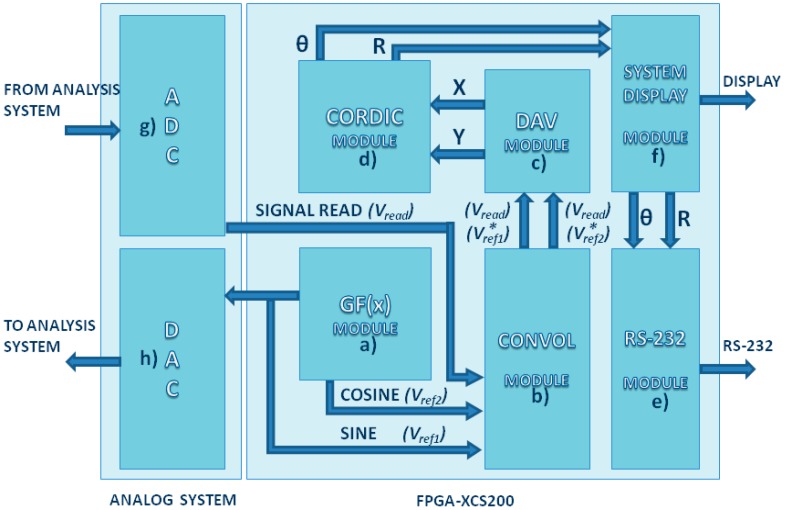
Complete block diagram: (**a**) Generation Function Module; (**b**) Convolution Module; (**c**) Dynamic Average Module; (**d**) CORDIC Module (**e**) RS-232 Module (**f**) System Display Module; (**g**) Analog to Digital Converter MAX1204; and (**h**) Digital to Analog Converter DAC0800

This section describes the hardware design for the developed device. Taking account the minimal requirements for low-frequency photoacoustic applications, it consists of the Function Generation, Convolution, Dynamic Average, the CORDIC (Coordinated Rotation Digital Computer) Algorithm, and Data Transmission modules. Each one of these modules was developed with reconfigurable hardware: a Spartan 3 FPGA model XCS200, from XILINX (San José, CA, USA)), was used. The complete block diagram may be seen in Figure 3. 

### 4.1. The Function Generation Module 

It is necessary to modulate the desired signal to allow its detection and to perform the LIA effect. For this reason, the developed device integrates a Function Generation Module. This module consists of two LUTs that digitally store a sine waveform. The module also allows digital selection of the appropriate offset, amplitude, and frequency for the experimental setup. As a result, it is necessary to generate a reference signal with a +90° shift on the first reference because the second LUT has a constant digital phase shift. Finally, digital data are transformed to an analog signal using a DAC; for this experimental setup, a DAC0800 from National Instrument was used, but the developed module may be implemented using different kinds of parallel DACs. 

### 4.2. Convolution Module 

This module consists of two digital multipliers, which are embedded in the FPGA chip. The first multiplier performs the multiplication of the read signal by the sine reference. The second one multiplies the acquired signal by the cosine reference. This module performs the “Dual-Phase” LIA effect and calculates amplitude and phase simultaneously. The read signal is converted from analog to digital using a MAX1204 from MAXIM (San José, CA, USA). 

### 4.3. Dynamic Average Module 

Unlike commercial LIAs, after the convolution operation is made, the developed device employs a Dynamic Average Module to remove AC signals and to obtain the DC signal (offset level from the input signal), which contains amplitude and phase variations of the input signal (Equations (1) and (2)). Commercial LIAs employs a low-pass filter instead of the presented module; however, this module offers the advantage that it can be implemented into full digital systems. It also performs like an exclusive DC filter, because it sums all the samples from the read signal, which corresponds to a cycle from reference frequency, and then divide it by the number of taken samples. This operation is calculated for the output, which is obtained from the convolutions of sine and cosine. 

### 4.4. The CORDIC Algorithm Module 

The CORDIC algorithm is an alternative to calculating *R* and *θ* from X and Y vectors (Equation (3)). CORDIC algorithm uses successive approximations to calculate *R* and *θ*, without the employment of complex operations, like square roots. The CORDIC algorithm was obtained from VHDL-GNU (Henderson, NV, USA) public license. 

### 4.5. Data Transmission Module 

This module permits the data transmission, via RS-232, to store the results in a computer, or any other storage compatible device, for future analysis. As the last module, this core was obtained from VHDL-GNU public license. 

### 4.6. The Calibration Process 

In order to ensure frequency stability of the Functions Generation Module, the designed device was calibrated using a Timer/Counter Analyzer CNT-91 50 ps/300 MHz (Rochester, NY, USA) from Pendulum, locked to the Primary Frequency Standard Cesium 5071A from Hewlett-Packard at “Time and Frequency Division—CENAM, Mexico.” For stability test, a 9 kHz frequency was selected from implemented device, an analog low pass filter was connected to the DAC output to avoid signal quantization. 

**Figure 4:**
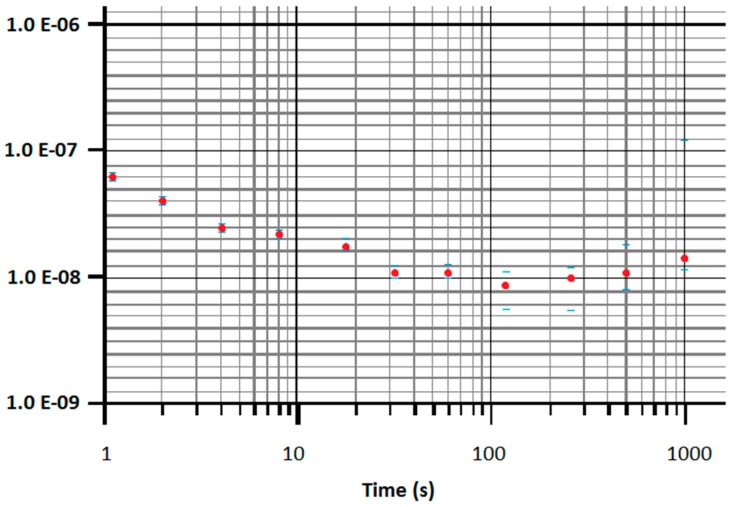
Results from stability test using Allan deviation as a stability analysis tool at 1–1000 s analysis windows.

Figure 4 shows results from stability test that used the Allan deviation as a stability analysis tool. It is important to mention that similar results at any desired frequency from 1 Hz to 10 kHz will be obtained. 

## 5. Results of Commercial and Designed LIAS Comparison 

**Figure 5:**
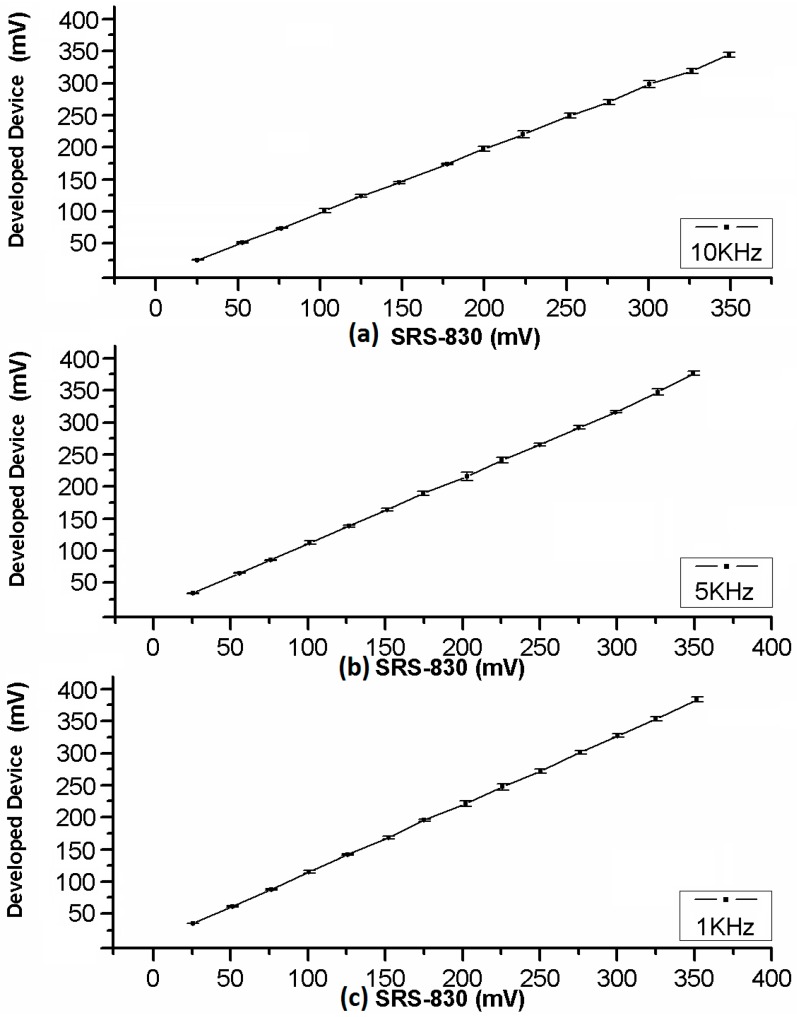
Amplitude detection response of the proposal *versus* SR830 LIA at different frequencies: (**a**) 10 kHz; (**b**) 5 kHz; and (**c**) 1 kHz.

A comparison between the commercial and the developed LIA is made. The analysis of both amplitude and phase detection responses were made using commercial Stanford Research model SR830 Lock-In (Standford Research Systems, Sunnyvale, CA, USA,) versus the proposal. The SR830 has features as: 1 mHz to 102.4 kHz range, >100 dB dynamic reserve, 5 ppm/°C stability, 0.01° phase resolution, Time constants from 10 µs to 30 ks, *etc*. The results shown in Figure 5 comprise the detection of a custom signal at different amplitude levels, from 25 to 350 mV at 25 mV steps for three different frequencies: 1 kHz, 5 kHz, and 10 kHz. The graphs show the level detected by the commercial LIA SR830 *versus* the amplitude level detected. The value is directly obtained by the developed device from the CORDIC module as instrumental function results, before converting it to an equivalent value according to detection levels. It is worth noticing that the small value of standard deviation, from obtained results at the different frequencies, demonstrates the repeatability of the measurements. 

In the case of phase shift measures, they were carried out by a shift from 0 to 360° with 15° steps for three different frequencies: 1 kHz, 5 kHz, and 10 kHz. The phase shift obtained by the commercial LIA SR830 is graphed *versus* the phase shift obtained by developed device. The small variation in the measurements is also reflected in the small value of the standard deviation. 

**Figure 6:**
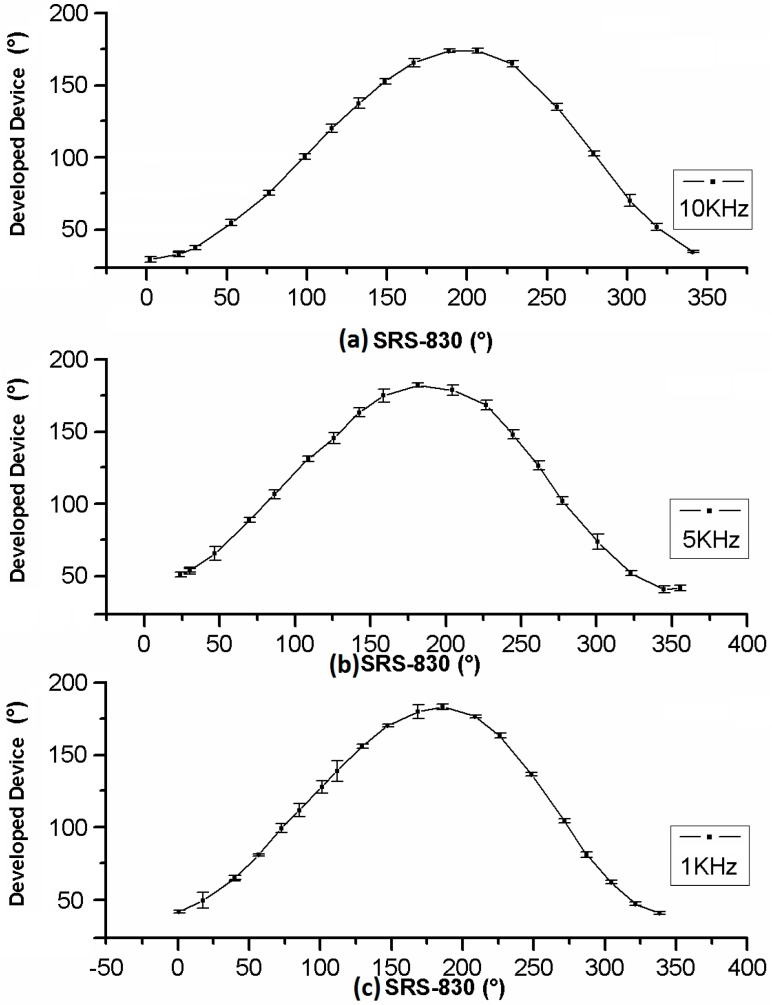
Phase Shift Detection response gotten by this work *versus* SR830 LIA at different frequencies: (**a**) 10 kHz; (**b**) 5 kHz; and (**c**) 1 kHz.

Obtained results were calculated from 10 different measurements in both cases, made by the commercial LIA SR830 and the developed device, showed on Figure 5 and Figure 6. 

## 6. Reduction of Space, Weight, and Cost of Implementation 

**Figure 7:**
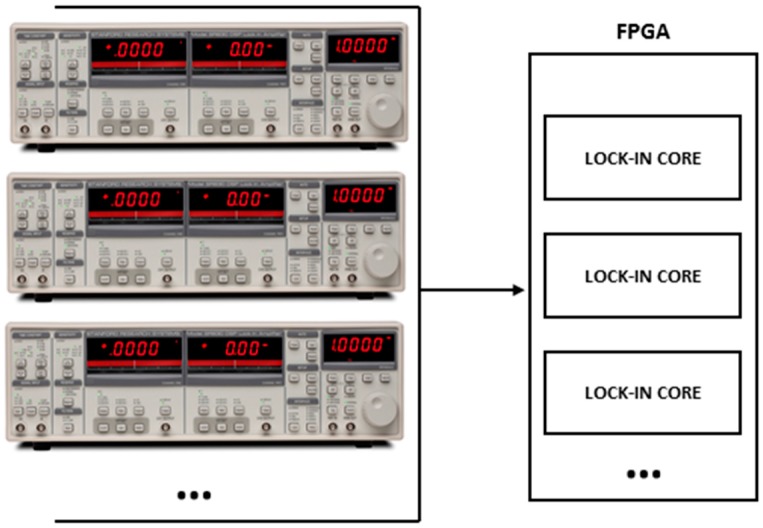
Many Lock-In Amplifier main functions (Developed Lock-In Core) can be implemented in the same FPGA circuit depending on the Logic Cells availability from the used circuit (SR-830 Lock-In Left Image from Thinksrs.com, (Sunnyvale, CA, USA).

**Figure 8:**
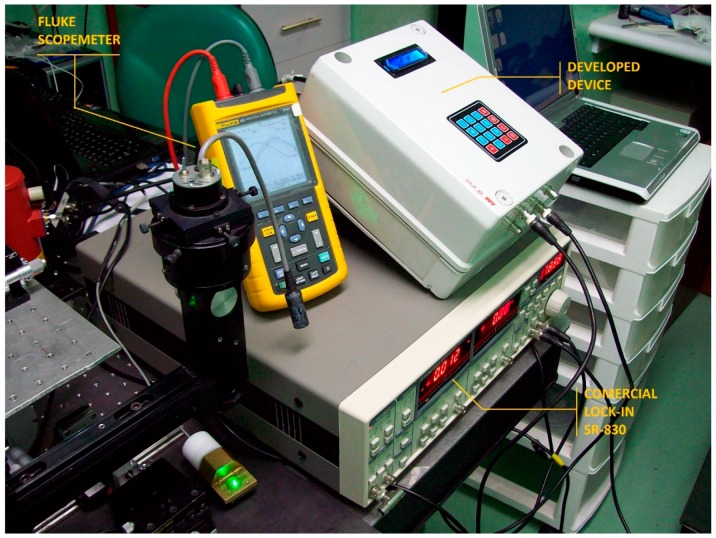
Developed core from the performed device can be implemented in the same FPGA circuit 10 times or more depending of the FPGA circuit used resulting in saving space, weight and cost implementation.

In addition to the obtained results in the calibration process, the developed device can be replicated many times in the same FPGA circuit depending on the capacity of logic cells from the FPGA circuit used, allowing a reduction of space, weight and cost of implementation for experiments that requires multiple FPGA Lock-In detectors (Figure 7). The comparison of the developed device containing the deloped core can be observed in Figure 8. This can allow at least ten times working in parallel the same main functions of the SR-830 Lock-In Amplifier from Stanford Research brand that show below, in a Spartan 3-XCS1000 that is five times larger than Spartan 3-XCS200 A Spartan 3-XCS200 that uses 41% of total logic cells is shown in Table 1. This is one advantage that can not be obtained using a different technology like DSP (Digital Signal Processor) or Microprocessors because those kinds of devices cannot operate in parallel mode, when the phase detection is important. 

In the developed device, some logic have a “Time Input Clock to Output from Digital Clock Manager (TICKOFDCM)”. Besides “Time Input Clock to Output (TICKOF)” does not use the Digital Clock Manager from the board. The latency is shown in Table 2.

The size, speed and the throughput of each divider are compared with units distributed by Xilinx. Obtaining the sizes in the Xilinx datasheet required the use of mapping options that have serious performance implications.

Thermal information, power consumption and electrical information are calculated by the Xpower Estimator (XPE) 11.1, provided by Xilinx, Inc. (San José, CA, USA), as shown Table 3, Table 4 and Table 5.

## 7. Conclusions

Allan deviation is shown in Figure 4; the stability is around 1.0e−9, and the windows used for Allan deviation were from 1 s to 100,000 s. In either frequency scan or time scan, this result is considered a good behavior because good frequency stability is necessary when determining results of experiments. 

The standard deviation in Figure 5 shows good stability and linearity at the full frequency operation range of amplitude detection. Equally, Figure 6 shows good stability and linearity at the full frequency range operation on Phase Shift Detection. 

Obtained results show good behavior from the developed device, and they demonstrate that it might be applicable to different kinds of photothermal experiments at low frequencies. In addition, due to the kind of technology used for its development, it might be able to be implemented as an online, permanent monitoring system, as part of a complex control system as either amplitude of phase detector, or any other low-frequency application that requires detecting amplitude or phase shift of a modulated signal with minimum cost and resource consumption. 

## Figures and Tables

**Table 1:** Total used logic cells for the proposed device implemented on a Spartan 3-XCS200.

Device Utilization Summary			
Logic Utilization	Used	Available	Utilization
Total Number Slice Registers	1611	3840	41%
Number used as Flip Flops	1601		
Number used as Latches	10		
Number of 4 inputs LUTs	2220	3840	57%
Number of occupied Slices	1370	1920	71%
Number of Slices containing only related logic	1370	1370	100%
Number of Slices containing unrelated logic	0	1370	0%
Total Number of inputs LUTs	2440	3840	63%
Number used as logic	2220		
Number used as a route-thru	208		
Nunmber used as Shift registers	16		
Number of bonded IO8s	57	173	32%
IO8 Latches	9		
Number of MULT 18 × 18s	4	12	33%
Number of GCLKs	6	8	75%
Total equivalent gate count fordesign	52,708		
Additional JTAG gate for IOBs	2736		

**Table 2:** Clock-to-Output Times for the XC3S200 and XC3S1000 from the developed device using 50 MHz integrated clock circuit from each board.

Symbol	Description	Condition	Device	Speed Grade (-4)	Units
TICKOFDCM	When Reading from output Flip-Flop (OFF), the time from when the transition on the Global Clock pin activates to data appearing at the Output pin. The DCM is in use.	LVCMOS25, 12 mA Output drive, Fast Slew rate whit DCM	XC3S200	1.75	ns
			XC3S1000	2.39	ns
TICKOF	When reading from OFF, the time from when the transition on the Global Clock pin activates to data appearing at the Output pin. The DCM is not in use.	LVCMOS25, 12 mA Output drive, Fast Slew rate with DCM	XC3S200	4.46	ns
			XC3S1000	4.59	ns

**Table 3:** Power summary of the proposal hardware.

Optimization	None
Data	Production
Quiescent(W)	0.098
Dynamic (W)	0.000
Total (W)	0.098

**Table 4:** Voltage summary of the proposal hardware.

Source	Voltage	Power (W)	ICC (A)	ICCQ (A)
VCCINT	1.2	0.043	0.000	0.036
VCCAUX	2.5	0.050	0.000	0.0020
VCCO 3.3	3.3	0.000	0.000	0.000
VCCO 2.5	2.5	0.005	0.000	0.002
VCCO 1.8	1.8	0.000	0.000	0.000
VCCO 1.5	1.5	0.000	0.000	0.000
VCCO 1.2	1.2	0.000	0.000	0.000

**Table 5:** Thermal summary of the proposal hardware.

Ambient Temp (°C)	25.0
Airflow (LFM)	250
ΘJA (°C/W)	19.7
Custom ΘJA	
Max Ambient (°C)	83.0
Junction Temp(°C)	26.9
